# The Effect of Algorithms on Copy Number Variant Detection

**DOI:** 10.1371/journal.pone.0014456

**Published:** 2010-12-30

**Authors:** Debby W. Tsuang, Steven P. Millard, Benjamin Ely, Peter Chi, Kenneth Wang, Wendy H. Raskind, Sulgi Kim, Zoran Brkanac, Chang-En Yu

**Affiliations:** 1 Department of Psychiatry and Behavioral Sciences, University of Washington, Seattle, Washington, United States of America; 2 Department of Veteran Affairs, VISN-20 Mental Illness Research, Education, and Clinical Center, Seattle, Washington, United States of America; 3 Department of Medicine, University of Washington School of Medicine, Seattle, Washington, United States of America; 4 Geriatric Research, Education, and Clinical Center, Veterans Affairs Puget Sound Health Care System, Seattle, Washington, United States of America; Innsbruck Medical University, Austria

## Abstract

**Background:**

The detection of copy number variants (CNVs) and the results of CNV-disease association studies rely on how CNVs are defined, and because array-based technologies can only infer CNVs, CNV-calling algorithms can produce vastly different findings. Several authors have noted the large-scale variability between CNV-detection methods, as well as the substantial false positive and false negative rates associated with those methods. In this study, we use variations of four common algorithms for CNV detection (PennCNV, QuantiSNP, HMMSeg, and cnvPartition) and two definitions of overlap (any overlap and an overlap of at least 40% of the smaller CNV) to illustrate the effects of varying algorithms and definitions of overlap on CNV discovery.

**Methodology and Principal Findings:**

We used a 56 K Illumina genotyping array enriched for CNV regions to generate hybridization intensities and allele frequencies for 48 Caucasian schizophrenia cases and 48 age-, ethnicity-, and gender-matched control subjects. No algorithm found a difference in CNV burden between the two groups. However, the total number of CNVs called ranged from 102 to 3,765 across algorithms. The mean CNV size ranged from 46 kb to 787 kb, and the average number of CNVs per subject ranged from 1 to 39. The number of novel CNVs not previously reported in normal subjects ranged from 0 to 212.

**Conclusions and Significance:**

Motivated by the availability of multiple publicly available genome-wide SNP arrays, investigators are conducting numerous analyses to identify putative additional CNVs in complex genetic disorders. However, the number of CNVs identified in array-based studies, and whether these CNVs are novel or valid, will depend on the algorithm(s) used. Thus, given the variety of methods used, there will be many false positives and false negatives. Both guidelines for the identification of CNVs inferred from high-density arrays and the establishment of a gold standard for validation of CNVs are needed.

## Introduction

Rapidly developing technologies such as chip array-based genotyping platforms have facilitated recent large-scale interrogation of the human genome. Many of these investigations have been successful in identifying specific single nucleotide polymorphisms (SNPs) associated with complex disorders [1], [2], but these investigations cannot identify all forms of genetic variation because they focus on the common SNPs [1]. The availability of densely spaced SNPs generated by genome-wide studies has also enabled the investigation of genome structural variations, such as copy number variants (CNVs). CNVs range in size from a few to several thousand base pairs (bp), and because they frequently affect gene dosage or structure, they are likely to have a biological impact. Furthermore, CNVs are likely enriched in genes encoding proteins related to human evolution and environmental adaptation [3], making CNVs ideal candidates for genetic susceptibility factors in complex disorders such as schizophrenia.

The presence of CNVs is inferred through array-based technologies using calling algorithms that can vary substantially and that can result in vastly different findings. These inferences are made based on hybridization intensities and allele frequencies. Large ratios of normalized intensities and/or higher than anticipated heterozygosity at specific genomic locations indicate excessive hybridization and suggest that a duplication, triplication, or other excess copies of the genomic region may exist, whereas small intensity ratios or long runs of homozygosity suggest that a deletion may be present.

The detection of previously undiscovered CNVs and the results of CNV-disease association studies rely on how CNVs are defined. Several authors have noted the large-scale variability between CNV-detection methods, as well as the substantial false positive and false negative rates associated with those methods (e.g., Winchester et al. [4], Zhang et al. [5]). For example, Winchester et al. [4] examined the results of a number of different SNP-based algorithms, including Birdsuite [6], Chromosome Copy Number Analysis Tool (CNAT) (www.Affymetrix.com), Genome Alteration Detection Algorithm (GADA; [7]), PennCNV [8], and QuantiSNP [9]. These algorithms were applied to CEPH sample NA12156 from HapMap, which was genotyped using both Illumina and Affymetrix arrays, as well as sequenced for structural variations using fosmid end-pair sequence (EPS) methods [10]. Whereas the EPS method detected a total of 638 CNV events, the number of events reported by the CNV algorithms ranged from 8 to 546, and the false positive rate (based on lack of overlap with the molecular method) ranged from 51% to 80%. Using the 299 events detected by Kidd et al. [10] on another CEPH sample (NA15510), Winchester et al. [4] found false negative rates ranging from 77% to 96%. Additionally, they compared consistency across algorithms and found that no pair of algorithms had greater than 60% concordance. Consequently, Winchester et al. [4] recommend using multiple algorithms and using software specific to the array platform that generated the data to identify CNVs.

Whether a CNV is newly discovered compared to the CNVs cataloged in a reference database depends on how overlap with previously discovered CNVs is defined. There are at least two issues to consider: (1) how to combine “overlapping” CNVs found in unique individuals into one CNV and (2) how to determine whether a potential newly discovered CNV overlaps with a reference CNV. Redon et al. [11] define CNV regions (CNVRs) as the union of locations where CNVs from multiple individuals have any (i.e., at least 1 bp) overlap, and Perry et al. [12] use this definition, as do Cooper et al. [13]. Redon et al. [11] also define independent juxtaposed CNVs according to the criterion that individual-specific CNVs must overlap by more than a threshold proportion (e.g., 40% of the length of *each* CNV) in order to be merged. In the context of identifying *de novo* CNVs in an individual that were not present in either parent, McCarroll et al. [14] use stringent criteria, joining CNVs across samples only if they overlap across at least 80% of their length. Wain et al.'s [15] definition of CNV loci is similar to Redon et al.'s independent juxtaposed CNVs except that the CNV locus is defined as the intersection (not the union) of the overlapping CNVs. Wain et al. [15] showed that the selection of overlap threshold for defining CNV loci affected the nominal significance level in a genome-wide association study of amyotrophic lateral sclerosis. However, the general effects of overlap definitions on identified CNVs have not been specifically compared across studies.

**Figure 1:**
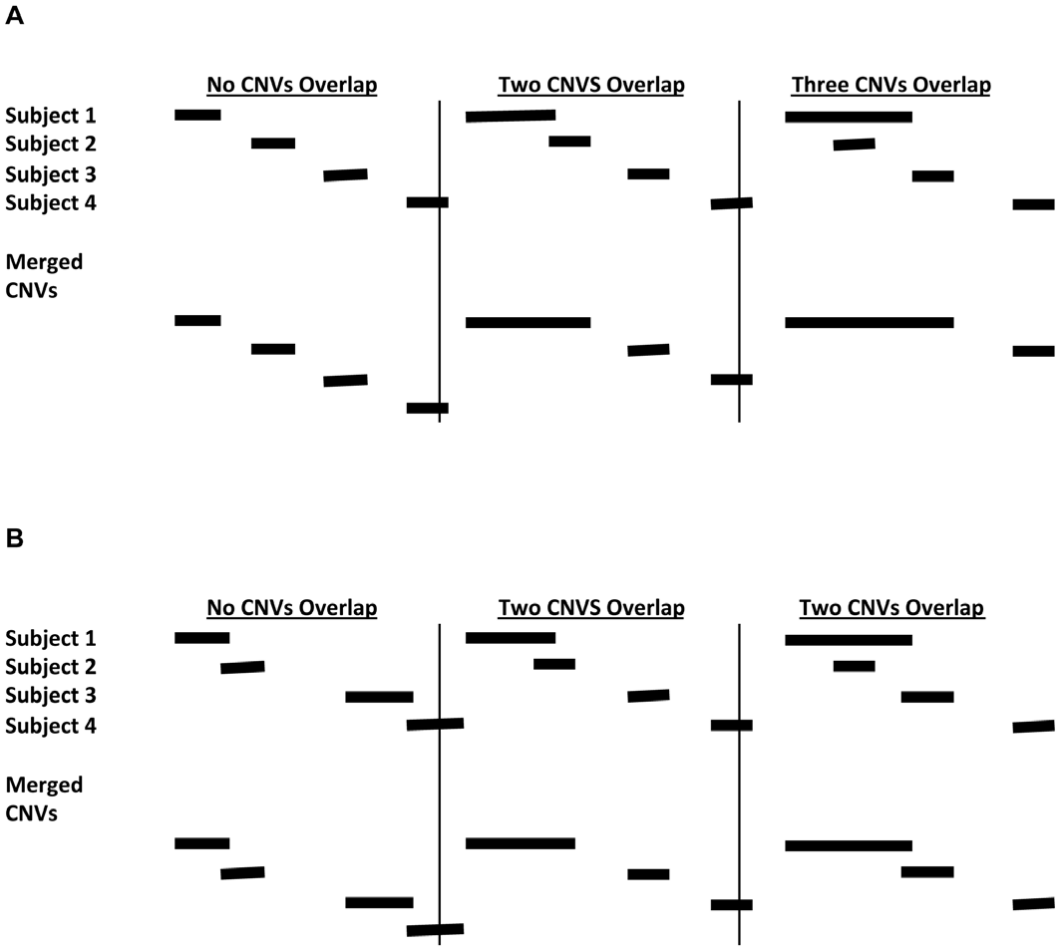
Illustration of how CNVs are merged based on the “any” overlap criterion (A) and the “40% either” overlap criterion (B). (A) CNVs that have any overlap are merged. The start position of the resulting CNV is defined to be the minimum base pair position of the overlapping CNVs, and the end position is defined to be the maximum base pair position of the overlapping CNVs. (B) CNVs are merged only if the length of overlap is at least 40% of the size of at least one of the CNVs. The start position of the resulting CNV is defined to be the minimum base pair position of the overlapping CNVs, and the end position is defined to be the maximum base pair position of the overlapping CNVs.

In this study, we use variations of four common algorithms for CNV detection (PennCNV [8], QuantiSNP [9], HMMSeg [16], and cnvPartition [17]) and two definitions of overlap (any overlap and an overlap of at least 40% of the smaller CNV; see Figure 1) to illustrate the effects of varying algorithms and definitions of overlap on CNV identification and discovery. Our initial sample included 50 Caucasian schizophrenia cases and 48 age-, ethnicity-, and gender-matched control subjects who were evaluated with a 56 K Illumina genotyping array enriched for CNV regions (deCODE; www.decode.com). Although no algorithm variation found a difference in CNV burden between the two groups (results not shown), we found substantial differences between the results generated by the algorithms for the number of CNVs, size of CNVs, CNVs per person, and whether or not we discovered novel CNVs.

## Results

### Merging CNVs Reported in Published Databases

As of March 2009, there were 29,292 CNVs reported in the literature from normal subjects [18], [19], [20], [21], [22], [23]. However, after merging CNVs across individuals and between studies, only 6,735 unique CNVs (i.e., CNVRs) exist when applying the “any” overlap criteria (Table 1). Of the 3,581 CNVs reported in the literature for schizophrenia subjects [20], [21], [22], [23], only 479 were not already reported for normal subjects, and of these, 418 are unique. Using the “40% either” overlap criterion instead of the “any” overlap criterion increases these numbers only slightly (Table 1). Supplemental Figures S1, S2, S3, S4 show summary statistics for the numbers and sizes of CNVs within merged CNV groups.

**Table 1:** Number of CNVs in Normal and SCZ databases based on the literature, by overlap algorithm.

Overlap Algorithm^1^	Merged CNVs in Normals^2^				CNVs in SCZ from Lit, Not Previously Discovered in Normals^3^				Merged Version of CNVs in SCZ from Lit, Not Previously Discovered in Normals			
	Loss	Gain	Both	Total	Loss	Gain	Both	Total	Loss	Gain	Both	Total
**Any**	4,007	1,273	1,455	6,735	135	344		479	119	293	6	418
**40% Either**	4,293	1,426	1,506	7,225	167	414		581	148	357	6	511

1 “Any” overlap means the CNVs share at least one base pair. “40% either” overlap means that the length of the overlap has to be at least 40% of the size of at least one of the CNVs.

2 Normals database contains 29,292 CNVs (9,538 gains; 18,983 losses; 771 both) before any kind of internal merging based on overlap is performed.

3 SCZ database contains 3,581 CNVs before omitting any CNVs that overlap with the CNVs in Normals database.

### Effect of Algorithms on Number of CNVs Detected

Table 2 shows the number of CNVs detected in 96 subjects (48 schizophrenia subjects and 48 control subjects) for each algorithm by type (loss versus gain; see Methods). The total number of CNVs detected ranged from 3,765 based on PennCNV alone to 102 based on requiring HMMSeg, cnvPartition with a 3-probe minimum, PennCNV, and QuantiSNP to all identify the same CNV. With the exception of the algorithms that involved cnvPartition with a 10-probe minimum, most of the detected CNVs were less than 100 kilobases (kb). For all the detected CNVs, losses were more common than gains, with the ratio ranging from 7-to-1 to 2-to-1. Again, with the exception of the algorithms that involved cnvPartition with a 10-probe minimum, most of the losses were less than 100 kb, whereas for all of the algorithms except PennCNV, most of the gains were greater than or equal to 100 kb. The average size of the detected CNVs ranged from 46 kb based on PennCNV alone to 787 kb based on cnvPartition with a 10-probe minimum (Table 3). The largest CNV found by PennCNV or HMMSeg alone was under 2 megabases (Mb), and the largest found by QuantiSNP alone was slightly under 5 Mb, but all other algorithms (all of which involved cnvPartition) detected a 10-Mb CNV. The average number of CNVs per person (Table 4) ranged from 39.2 based on PennCNV alone to 1.1 based on requiring two algorithms (HMMSeg and cnvPartition with a 10-probe minimum) or four algorithms (HMMSeg, cnvPartition with a 3-probe minimum, PennCNV, and QuantiSNP) to all identify the same CNV. Note that for six of the algorithms, there were several subjects with no CNVs detected. For Tables 2, 3, and 4, the results of algorithms that involve determining overlap (the last four rows listed in each table) were the same regardless of which definition of overlap was used (“any” versus “40% either”; see the Methods section and Figure 1). Supplemental Figure S5 shows the range of sizes of overlapping CNVs (within individual and chromosome) by chromosome for CNVs identified by requiring HMMSeg, cnvPartition with a 3-probe minimum, PennCNV, and QuantiSNP to all identify the CNV.

**Table 2:** Number of CNVs detected in 96 subjects by each algorithm.

	All CNVs			CNVs <100 kb			CNVs ≥100 kb		
Algorithm	Loss	Gain	Total	Loss	Gain	Total	Loss	Gain	Total
PennCNV^1^	2,531	1,234	3,765	2,280	966	3,246	251	268	519
HMMSeg^2^	664	302	966	584	27	611	80	275	355
cnvPartition with 3 Probes^3^	590	103	693	432	28	460	158	75	233
cnvPartition with 5 Probes^3^	427	87	514	289	12	301	138	75	213
cnvPartition with 10 Probes^3^	175	75	250	93	4	97	82	71	153
QuantiSNP^4^	159	81	240	117	21	138	42	60	102
HMMSeg & cnvPartition with 3 Probes^5^	262	37	299	215	2	217	47	35	82
HMMSeg & cnvPartition with 5 Probes^5^	172	37	209	129	2	131	43	35	78
HMMSeg & cnvPartition with 10 Probes^5^	71	34	105	35	1	36	36	33	69
HMMSeg & cnvPartition with 3 Probes & PennCNV & QuantiSNP^6^	87	15	102	56	2	58	31	13	44

1 Default settings, then CNVs <10 bp omitted.

2 HMMSeg using Cooper et al. [13] implementation.

3 Default settings, except minimum number of probes required to identify that a CNV was varied.

4 Default settings, then CNVs with Log Bayes Factor <30 omitted.

5 Only CNVs identified by both HMMSeg and cnvPartition that overlap are included.

6 Only CNVs identified by HMMSeg, cnvPartition, PennCNV, and QuantiSNP that overlap are included.

**Table 3:** Size of CNVs (kb) detected in 96 subjects by each algorithm.

	All CNVs				CNVs <100 kb				CNVs ≥100 kb			
Algorithm	Mean	SD	Min	Max	Mean	SD	Min	Max	Mean	SD	Min	Max
PennCNV	46	105	0.003	1,623	17	25	0.003	100	226	195	100	1,623
HMMSeg	126	215	1	1,751	16	25	1	100	316	260	100	1,751
cnvPartition with 3 Probes	345	998	0.1	10,283	31	33	0.1	99	966	1,544	102	10,283
cnvPartition with 5 Probes	443	1,138	1	10,283	40	35	1	99	1,013	1,605	102	10,283
cnvPartition with 10 Probes	787	1,550	8	10,283	30	17	8	94	1,266	1,827	103	10,283
QuantiSNP	410	849	1	4,733	39	36	1	99	911	1,123	100	4,733
HMMSeg & cnvPartition with 3 Probes	247	801	1	10,283	28	32	1	99	827	1,375	103	10,283
HMMSeg & cnvPartition with 5 Probes	344	942	2	10,283	38	36	2	99	857	1,403	103	10,283
HMMSeg & cnvPartition with 10 Probes	607	1,270	8	10,283	32	10	8	52	907	1,484	121	10,283
HMMSeg & cnvPartition with 3 Probes & PennCNV & QuantiSNP	345	1,129	2	10,283	45	43	2	99	740	1,646	110	10,283

See Table 2 for explanation of algorithms.

**Table 4:** Number of CNVs per person detected in 96 subjects by each algorithm.

	All CNVs				CNVs <100 kb				CNVs ≥100 kb			
Algorithm	Mean	SD	Min	Max	Mean	SD	Min	Max	Mean	SD	Min	Max
PennCNV	39.2	11.2	14	78	33.8	9.0	14	60	5.4	4.8	0	30
HMMSeg	10.1	3.4	3	26	6.4	1.9	3	12	3.7	2.7	0	17
cnvPartition-with 3 Probes	7.2	3.6	1	23	4.8	2.3	1	12	2.4	2.7	0	20
cnvPartition with 5 Probes	5.4	3.1	1	23	3.1	1.7	0	7	2.2	2.6	0	20
cnvPartition with 10 Probes	2.6	2.6	0^a^	21	1.0	0.9	0	3	1.6	2.3	0	19
QuantiSNP	2.5	2.7	0^b^	20	1.4	1.2	0	5	1.1	2.5	0	19
HMMSeg & cnvPartition-with 3 Probes	3.1	1.7	0^c^	8	2.3	1.4	0	7	0.9	1.1	0	5
HMMSeg & cnvPartition with 5 Probes	2.2	1.5	0^d^	6	1.4	1.1	0	5	0.8	1.1	0	5
HMMSeg & cnvPartition with 10 Probes	1.1	1.2	0^e^	5	0.4	0.5	0	2	0.7	1.0	0	5
HMMSeg & cnvPartition with 3 Probes & PennCNV & QuantiSNP	1.1	1.1	0^f^	5	0.6	0.8	0	3	0.5	0.8	0	4

See Table 2 for explanation of algorithms.

a 4 control and 5 schizophrenia subjects with no identified CNVs.

b 6 control and 6 schizophrenia subjects with no identified CNVs.

c 1 control and 2 schizophrenia subjects with no identified CNVs.

d 5 control and 5 schizophrenia subjects with no identified CNVs.

e 15 control and 19 schizophrenia subjects with no identified CNVs.

f 19 control and 17 schizophrenia subjects with no identified CNVs.

### Effect of Algorithms on Number of “Newly Discovered” CNVs

**Figure 2:**
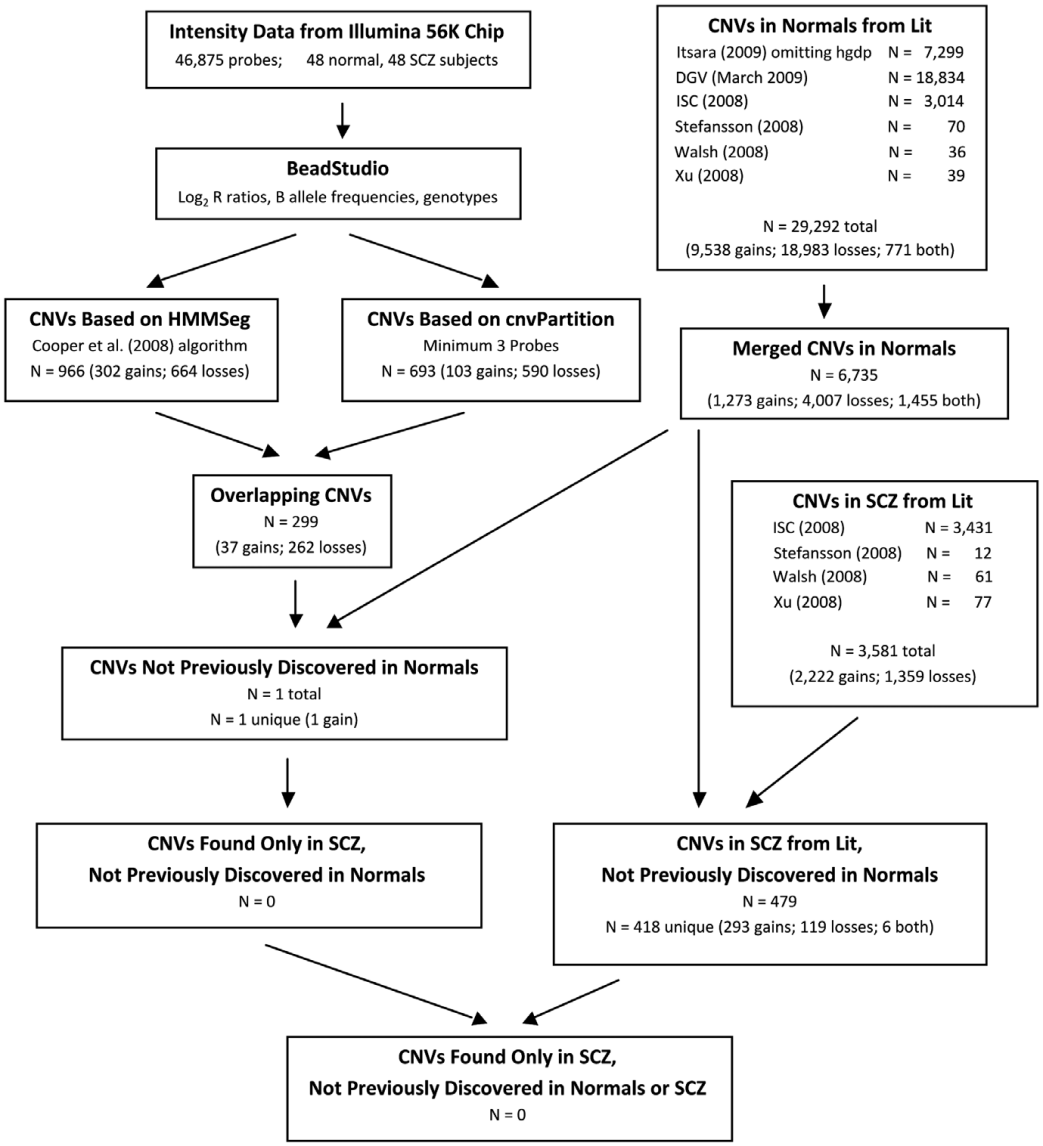
Process Flow Chart based on overlap between HMMSeg and cnvPartition (3-probe minimum). Numbers are based on the “any” overlap criterion. All data on sex chromosomes have been omitted. Gains are compared only to gains or both, and losses are compared only to losses or both.

Figure 2, supplemental Figures S6, S7, S8, S9, S10, and Tables 5 and 6 demonstrate that we found widely varying results depending on the algorithm(s) used. Figure 2 demonstrates that requiring both cnvPartition with a 3-probe minimum and HMMSeg to identify CNVs using the “any” overlap criterion results in finding one CNV not previously reported in the literature for normal subjects (a gain at genomic locations 25046920–25130278 on chromosome 8). However, this CNV was found in a control subject, thus no novel CNVs were discovered in schizophrenia subjects. Using HMMSeg alone with the “any” overlap criterion, we found two novel CNVs (both gains) in schizophrenia subjects (supplemental Figure S6), whereas using only cnvPartition with a 3-probe minimum with the “any” overlap criterion, we found three novel CNVs (all losses) in schizophrenia subjects (supplemental Figure S7). Thus, using either algorithm alone resulted in discovering novel CNVs in schizophrenia subjects (although not of the same type), but using the criterion that both algorithms must identify the same CNV resulted in the discovery of no novel CNVs in schizophrenia subjects.

**Table 5:** “Newly discovered” CNVs detected by each algorithm based on 48 normal and 48 schizophrenia subjects, using the “any” overlap criterion.

Algorithm	CNVs Not Previously Discovered in Normals				CNVs Found Only in SCZ, Not Previously Discovered in Normals				CNVs Found Only in SCZ, Not Previously Discovered in Normals or SCZ			
	Loss	Gain	Both	Total	Loss	Gain	Both	Total	Loss	Gain	Both	Total
PennCNV	72	84	33	189	24	31	2	57	24	29	2	55
HMMSeg	4	6		10		2		2		2		2
cnvPartition with 3 Probes	12	2		14	3			3	3			3
cnvPartition with 5 Probes	10	1		11	4			4	4			4
cnvPartition with 10 Probes	2			2	1			1	1			1
QuantiSNP		1		1				0				0
HMMSeg & cnvPartition with 3 Probes		1		1				0				0
HMMSeg & cnvPartition with 5 Probes		1		1				0				0
HMMSeg & cnvPartition with 10 Probes				0				0				0
HMMSeg & cnvPartition with 3 Probes & PennCNV & QuantiSNP				1				0				0

See Table 2 for explanation of algorithms.

**Table 6:** “Newly discovered” CNVs detected by each algorithm based on 48 normal and 48 schizophrenia subjects, using the “40% either” overlap criterion.

Algorithm	CNVs Not Previously Discovered in Normals				CNVs Found Only in SCZ, Not Previously Discovered in Normals				CNVs Found Only in SCZ, Not Previously Discovered in Normals or SCZ			
	Loss	Gain	Both	Total	Loss	Gain	Both	Total	Loss	Gain	Both	Total
PennCNV	83	98	31	212	30	34	2	66	30	28	2	60
HMMSeg	6	10		16	1	4		5	1	3		4
cnvPartition with 3 Probes	14	4		18	3			3	3			3
cnvPartition with 5 Probes	10	3		13	4			4	4			4
cnvPartition with 10 Probes	2	1		3	1			1	1			1
QuantiSNP		1		1				0				0
HMMSeg & cnvPartition with 3 Probes	1	2		3	1			1				0
HMMSeg & cnvPartition with 5 Probes		2		2				0				0
HMMSeg & cnvPartition with 10 Probes		1		1				0				0
HMMSeg & cnvPartition with 3 Probes & PennCNV & QuantiSNP		1		1				0				0

See Table 2 for explanation of algorithms.

Tables 5 and 6 show results for all ten algorithms. Using the “any” overlap criterion, the number of novel CNVs compared to previously published normal databases ranged from 189 to 0 over the ten algorithms, and the number of novel CNVs found in our schizophrenia subjects that were not previously reported in either normal or schizophrenia subjects ranged from 55 to 0 (Table 5). Using the “40% either” overlap criterion resulted in a slightly higher number of novel CNVs (Table 6). The coordinates of CNVs not previously published in databases of normal subjects are given in supplemental Datasets S1–S10 in File S1.

## Discussion

Multiple recent studies have used new whole-genome genotyping methods to discover structural variations in the DNA segments of normal subjects and subjects with a variety of disorders. The identification of novel rare CNVs in autism (*NRXN1*, *SHANK3*, and *CNTNAP2* [24]) and schizophrenia [21] has generated much excitement. These CNVs range in size from a kb to several Mb. The majority of these CNVs are thought to be rare, highly penetrant, and found in only a small number of individuals (e.g., <1% of subjects with schizophrenia). However, the role of specific genes within these CNVs that are associated with schizophrenia remains unknown.

We, Zhang et al. [5], and others [4] demonstrate that the number of CNVs identified depends on the algorithm(s) utilized. Because CNVs are inferred from observed intensity data instead of being directly called, as is the case for SNP genotypes, Winchester et al. [4] recommend using two calling algorithms instead of just one. However, although the net effect of this strategy decreases the false positive rate, it also increases the false negative rate. Furthermore, Carter [25] notes that it is inevitable that any hybridization studies will generate false positive and false negative results, regardless of how the data are analyzed. It is particularly important that these two rates are assessed in any study that uses SNP arrays for CNV detection, as high false positive rates will lead to publicly available databases becoming populated with regions incorrectly called as CNVs. However, without a true gold standard (e.g., full-genome sequencing), the false positive and false negative rates of any particular algorithm or combination of algorithms are impossible to estimate. Many of the regions in CNV databases today will prove to be false discoveries, particularly loci that have not been validated independently or are not replicated between studies [25]. Finally, in hybridization studies, standardized measures of uncertainty (e.g., confidence intervals) are unavailable in the literature due to unknown statistical properties of the algorithms (e.g., some published results were derived from algorithms that include manual inspection), inconsistent definitions of a “reference” genome, and a lack of commonly implemented gold standards.

Despite multiple reports of associations between specific CNVs and a disease, it is important to note that CNVs are also commonly found in normal individuals [26], and the presence of a CNV does not necessarily indicate that it is related to the disease phenotype [27]. Therefore, the selection of the appropriate reference database of CNVs found in “normal” individuals is critical. With a few exceptions, the majority of previous publications do not discuss in detail the definition of “novel” CNVs, that is, CNVs that were not previously found in normal reference databases or literature. We demonstrate that the choice of algorithm and overlap criteria affects how many (if any) CNVs are found that have not been previously reported in the literature.

Limitations of this study include the relatively low array density of the 56 K chip we used compared to current commonly used higher density chips. However, both Winchester et al. [4], who used Illumina 1 M and Affymetrix 6.0 arrays, and Zhang et al. [5], who used the Affymetrix 6.0 array, also found large variability in the number of CNVs detected depending on the algorithm used. Another limitation of our study is that because we did not find novel CNVs associated with schizophrenia, we did not proceed with molecular validation. However, the main point of this study was to demonstrate the marked variability in putative CNV detection between algorithms, not to demonstrate whether any of the CNVs were in fact valid.

In summary, both better guidelines for identifying CNVs using high-density arrays and a gold standard for validation of CNVs are needed. Although the availability of high-density SNP arrays increases the opportunity for discovery of novel genetic variants, much caution is necessary to establish CNV–disease associations. In general, molecular validation is necessary to confirm the presence of CNVs. Ultimately, the role of putative “disease-causing” gene(s) that are disrupted within CNVs will require additional confirmatory molecular genetic and molecular biologic studies. The application of the various algorithms to datasets that do not include molecular validations will generate many false positives. Issues of sensitivity and specificity will need to be further evaluated with next-generation sequencing (such as genomic resequencing data from the 1000 Genome Project; http://browser.1000genomes.org/index.html). The availability of genome-wide sequencing data will help to establish consensus guidelines for the identification and validation of true CNVs.

## Methods

### Ethics Statement

This study was conducted according to the principles expressed in the Declaration of Helsinki. Both the Consortium on the Genetics of Schizophrenia (COGS) and the University of Washington (UW) Alzheimer's Disease Research Center (ADRC) studies were approved by both the UW institutional review board and the VA Puget Sound Health Care System institutional review board. All subjects provided written informed consent for the collection of samples and subsequent analyses.

### Subjects

We recruited 50 schizophrenia subjects and 20 control subjects between 2003 and 2008 as part of the NIH-funded COGS [28]. Schizophrenia subjects met the DSM-IV-TR criteria for schizophrenia via the administration of the Diagnostic Interview for Genetic Studies (DIGS; [29]) and the Family Interview for Genetic Studies (FIGS; [30]). The ascertainment and screening procedures and inclusion/exclusion criteria are discussed in detail by Calkins et al. [28]. Control subjects did not meet DSM criteria for schizophrenia or other psychotic disorders and did not have a family history of schizophrenia or other psychotic disorders. An additional 28 control subjects were obtained from the UW ADRC [31].

Of the 50 schizophrenia subjects, DNA from 1 subject was not genotyped due to poor DNA quality, and 1 subject was omitted from the analysis due to a substantially lower call rate compared to all of the other samples. The 48 remaining schizophrenia subjects had a mean age of forty-one years (SD 12), and 9 (19%) were female, compared with a mean age of forty-four years (SD 13) for the 48 control subjects, of whom 9 (19%) were female.

### SNP Genotyping

We prepared DNA from peripheral blood samples using standard protocols in order to avoid artifacts related to transformation and cell culture. We submitted 98 samples (50 schizophrenia subjects and 48 age-, ethnicity-, and gender-matched controls) to deCODE Genetics (www.decode.com) for genotyping. Because of our specific interest in CNVs, we chose to genotype our samples using the 56 K CNV-enriched deCODE-Illumina BeadChip array. This platform contains 52,167 markers: 34,965 polymorphic markers (67%) and 17,202 nonpolymorphic markers (33%). After excluding markers on sex chromosomes, there were 46,875 markers, including 32,159 polymorphic markers (69%) and 14,716 nonpolymorphic markers (31%), with an average distance of 59 kb between markers (SD  = 228 kb, range  = 1 to 21,470 kb).

### Identifying CNVs

We received SNP intensity data on genotypes with a call rate of greater than 95% from deCODE and then read these into Illumina's BeadStudio software (version 3.1.3.0; Genotyping version 3.3.7; Illumina Genome Viewer 3.2.9; www.illumina.com). Intensities were normalized by forming clusters using the raw data (as opposed to forming clusters using some external source, such as HapMap samples). The resulting log_2_ R ratios (LRR) and B-allele frequencies (BAF) [32] were used to identify CNVs on autosomes for each subject. We used variations of four algorithms for CNV detection: PennCNV [8] (May 1, 2010), QuantiSNP [9] (version 2.3), Cooper et al.'s [13] implementation of the Hidden Markov Segmentation Model (HMMSeg, [16]), and cnvPartition [17] (version 1.2.1). For PennCNV, we used the default settings, then omitted CNVs less than 10 bp. For QuantiSNP, we used the default settings, and then, following the advice of the documentation, we omitted CNVs with a Log Bayes Factor less than 30. For cnvPartition, we used the default settings, except we varied the minimum number of consecutive probes necessary to define a CNV (3, 5, or 10). For HMMSeg, for homozygous deletion (loss) predictions, we required events to be at least 3 probes and 1 kb in length with an average LRR value less than −1. For hemizygous deletion events, we required at least 10 probes and 1 kb in length with an average LRR value <−0.25, and we required the proportion of heterozygous SNP calls to be less than 10%. For amplification events (gains), we required a minimum of 10 probes and 1 kb in length, LRR values of greater than 0.25, and BAF deviation values at heterozygous SNPs greater than 0.05. For all results except those from HMMSeg, none of the CNVs spanned the centromere (coordinates obtained from UCSC genome browser database; http://genome.ucsc.edu). One CNV identified by HMMSeg that spanned the centromere was split into two separate CNVs on either side of the centromere region.

Besides these six different algorithms (PennCNV, QuantiSNP, HMMSeg, and cnvPartition with a 3-, 5-, or 10-probe minimum), we also looked at results where CNVs were required to be identified by both the HMMSeg algorithm and the cnvPartition algorithm (3-, 5-, or 10-probe minimum) for each subject/chromosome combination. We also looked at results where CNVs were required to be identified by HMMSeg, cnvPartition with a 3-probe minimum, PennCNV, and QuantiSNP. In these instances, the CNVs identified by the two or four algorithms had to overlap; the start position of the resulting CNV was defined as the minimum bp position of the two overlapping CNVs, and the end position was defined as the maximum bp position of the overlapping CNVs. We used two different methods to determine whether two CNVs overlapped: (1) any overlap and (2) a condition where the length of the overlap had to be at least 40% of the size of at least one of the CNVs (denoted as the “40% either” criterion). Figure 1 illustrates these two different methods. In all cases, losses were compared only with losses, and gains were compared only with gains.

### Finding CNVs Previously Unreported in the Literature

To determine whether any of the CNVs we discovered in our schizophrenia subjects had not yet been reported in the literature and did not appear in our own normal control subjects, we first compared all of our CNVs (from both our normal control and schizophrenia subjects) to a database constructed from CNVs that had been reported in the literature for normal subjects. We then disregarded the CNVs we had discovered that were in this “normals” database and/or were present in our normal control subjects. To determine whether we discovered any CNVs in our schizophrenia subjects that had not been previously reported in either normal or schizophrenia subjects and that were not present in our own normal control subjects, we compared the remaining CNVs in our schizophrenia subjects to a database constructed from CNVs that had been reported in the literature for schizophrenia subjects.

#### Constructing the Database of CNVs in Normal Subjects from the Literature

To construct the database of CNVs that have been reported in the literature for normal subjects, we initially combined CNVs from six sources: Itsara et al. [19], Database of Genomic Variants (DGV; http://projects.tcag.ca/variation/)[18], ISC [20], Stefansson et al. [21], Walsh et al. [22], and Xu et al. [23]. CNVs reported on X and Y chromosomes were omitted. CNVs reported in Itsara et al. [19] and ISC [20] were translated from hg17 to hg18 using LiftOver (http://genome.ucsc.edu/cgi-bin/hgLiftOver). CNVs reported by Itsara et al. [19] from the hgdp study were omitted because subjects had neurological conditions. CNVs reported in Database of Genomic Variants (DGV; http://projects.tcag.ca/variation/) that were less than 10 bp were omitted, as were CNVs for which both gain and loss were reported as blank or 0.

We denoted this database “CNVs in Normals from Lit” (see Figure 2). This database contained CNVs that were reported as gains and losses, as well as some CNVs that were reported as both gains and losses (denoted “both”). We then merged overlapping CNVs to create a set of unique CNVs, and we denoted this database “Merged CNVs in Normals.” When determining whether CNVs overlapped, we compared gains only to gains or both, and we compared losses only to losses or both. We used our two different definitions of overlap (“any” and “40% either”) to produce two distinct databases.

#### Constructing the Database of CNVs Not Previously Discovered in Normal Subjects

To construct a database of CNVs that we had discovered that were not previously reported in normal subjects, we compared our CNVs with the “Merged CNVs in Normals” database and kept only CNVs that did not overlap. Again, we compared gains only to gains or both, and we compared losses only to losses or both. We denoted this database “CNVs Not Previously Discovered in Normals” (see Figure 2). We used our two different definitions of overlap to produce two distinct databases. From these remaining CNVs, we constructed a new database by keeping only those CNVs that were present in our schizophrenia subjects and not present in our normal control subjects; we denoted this database “CNVs Found Only in SCZ, Not Previously Discovered in Normals.”

#### Constructing the Database of CNVs in Schizophrenia Subjects from the Literature

To construct the database of CNVs that had been reported in the literature for schizophrenia subjects, we initially combined CNVs from four sources [20], [22]. We denoted this database “CNVs in SCZ from Lit” (see Figure 2). We then compared these CNVs with the CNVs in the “Merged CNVs in Normals” database and kept only CNVs that did not overlap. Again, we compared gains only to gains or both, and we compared losses only to losses or both. We denoted this database “CNVs in SCZ from Lit, Not Previously Discovered in Normals.” We used our two different definitions of overlap to produce two distinct databases.

#### Constructing the Database of CNVs Found Only in Schizophrenia Subjects and Not Previously Reported in Either Normal or Schizophrenia Subjects

To determine whether we had discovered any CNVs in our schizophrenia subjects that had not been previously reported in either normal or schizophrenia subjects, we compared the CNVs in the database “CNVs Found Only in SCZ, Not Previously Discovered in Normals” to the CNVs in the merged version of the database “CNVs in SCZ from Lit, Not Previously Discovered in Normals.” Again, we compared gains only to gains or both, and we compared losses only to losses or both. We denoted this database “CNVs Found Only in SCZ, Not Previously Discovered in Normals or SCZ.” We used our two different definitions of overlap to produce two distinct databases.

### Statistical Analysis

All data manipulation and statistical computations using the results of the CNV analyses were done in R version 2.8.1 [33].

## Supporting Information

Figure S1 Mean, Min, Max, and Range of CNV sizes within merged CNV Groups vs. number of CNVs in the group, using the “any” overlap criterion, for CNVs reported in the literature for normal subjects.
(2.80 MB TIF)

Figure S2 Mean, Min, Max, and Range of CNV sizes within merged CNV groups vs. number of CNVs in the group, using the “40% either” overlap criterion, for CNVs reported in the literature for normal subjects.
(2.80 MB TIF)

Figure S3 Number of CNVs per chromosome for CNVs reported in the literature for normal subjects, as well as number of CNVs based on merging overlapping CNVs.
(2.80 MB TIF)

Figure S4 Distribution of CNV size for CNVs reported in the literature for normal subjects, as well as distribution of CNV size based on merging overlapping CNVs.
(2.80 MB TIF)

Figure S5 Range of sizes of overlapping CNVs (within individual and chromosome) vs. chromosome for CNVs identified by requiring HMMSeg, cnvPartition with a 3-probe minimum, PennCNV, and QuantiSNP to all identify the CNV. N = 102 CNV groups (15 gains; 87 losses).
(2.80 MB TIF)

Figure S6 Process Flow Chart based on HMMSeg alone.
(2.80 MB TIF)

Figure S7 Process Flow Chart based on cnvPartition (3-probe minimum) alone.
(2.80 MB TIF)

Figure S8 Process Flow Chart based on PennCNV alone.
(2.80 MB TIF)

Figure S9 Process Flow Chart based on QuantiSNP alone.
(2.80 MB TIF)

Figure S10 Process Flow Chart based on overlap between HMMSeg, cnvPartition (3-probe minimum), PennCNV, and QuantiSNP.
(2.80 MB TIF)

File S1 Datasets S1–S10. Coordinates of CNVs not previously published in databases of normal subjects.
(0.24 MB XLS)
