# Supplementary figures and images for: The Effect of Algorithms on Copy Number Variant Detection
Source: PLoS One. 2010 Dec 30;5(12):e14456. doi: 10.1371/journal.pone.0014456 (PMC3012691; doi:10.1371/journal.pone.0014456)

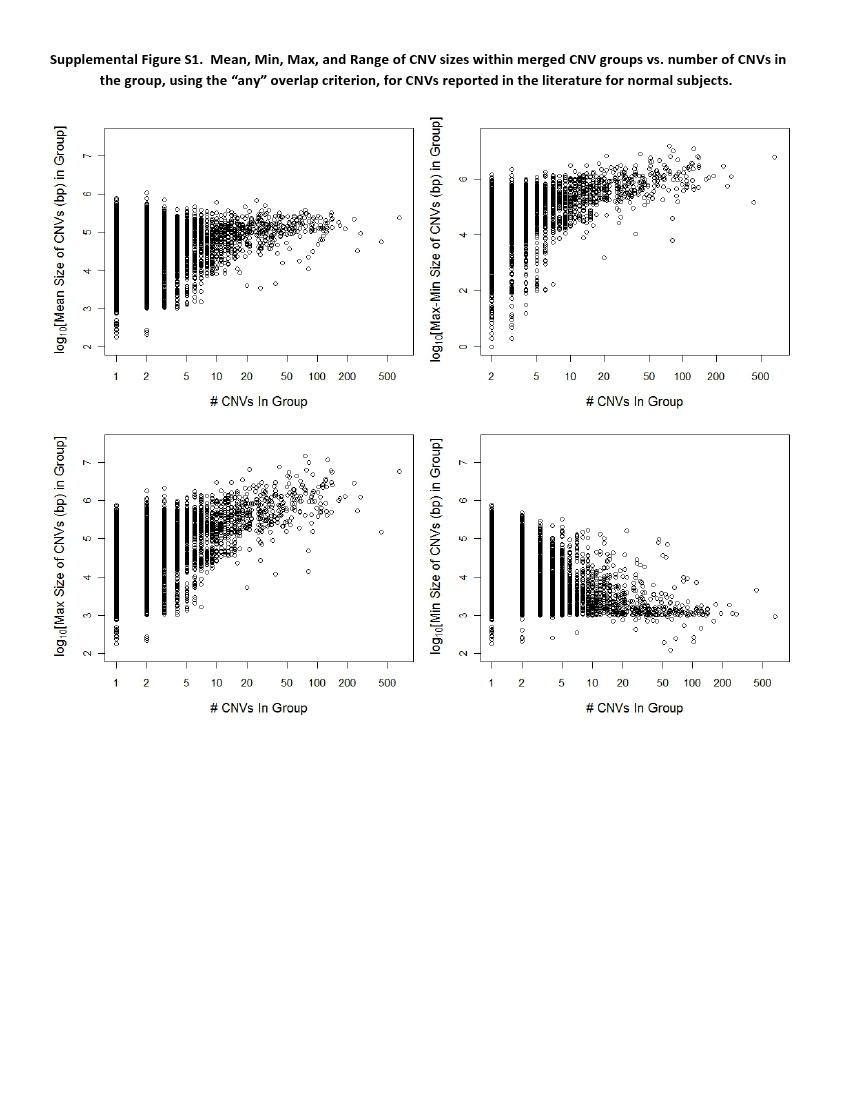

Supplement: Figure S1 — Mean, Min, Max, and Range of CNV sizes within merged CNV Groups vs. number of CNVs in the group, using the “any” overlap criterion, for CNVs reported in the literature for normal subjects. (2.80 MB TIF) [file pone.0014456.s001.tif]

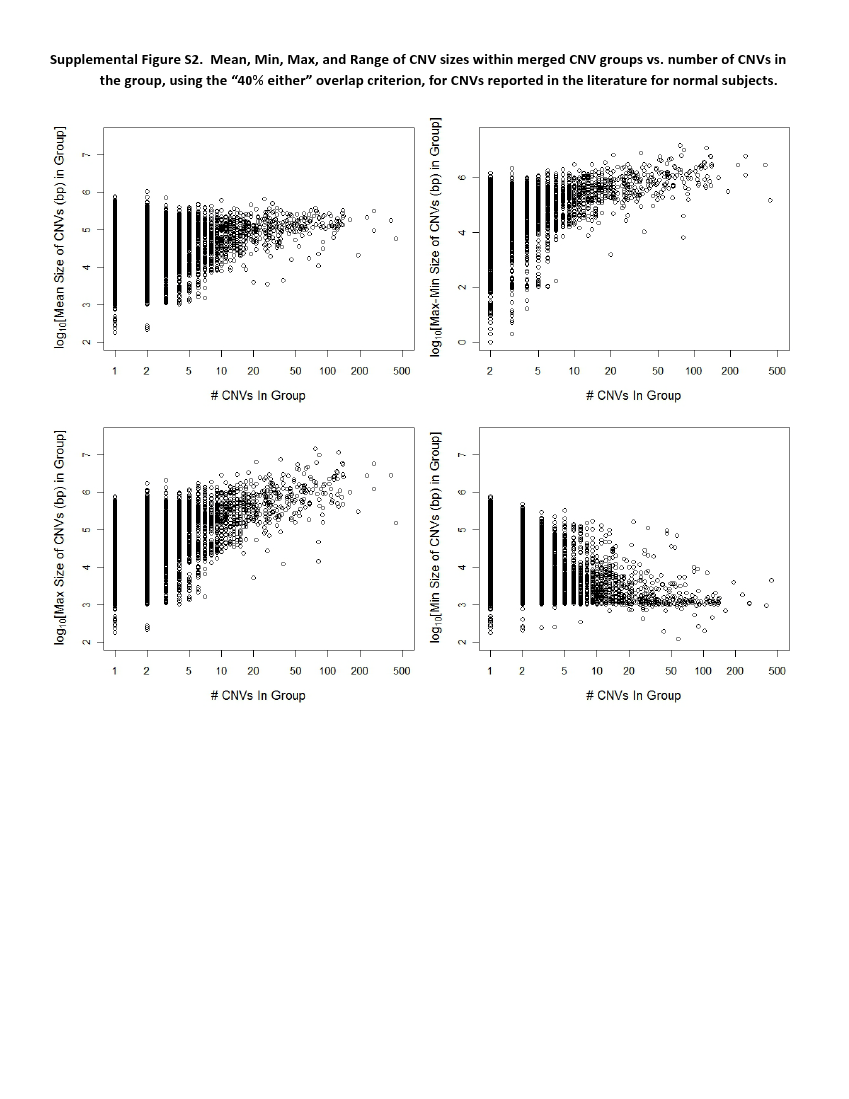

Supplement: Figure S2 — Mean, Min, Max, and Range of CNV sizes within merged CNV groups vs. number of CNVs in the group, using the “40% either” overlap criterion, for CNVs reported in the literature for normal subjects. (2.80 MB TIF) [file pone.0014456.s002.tif]

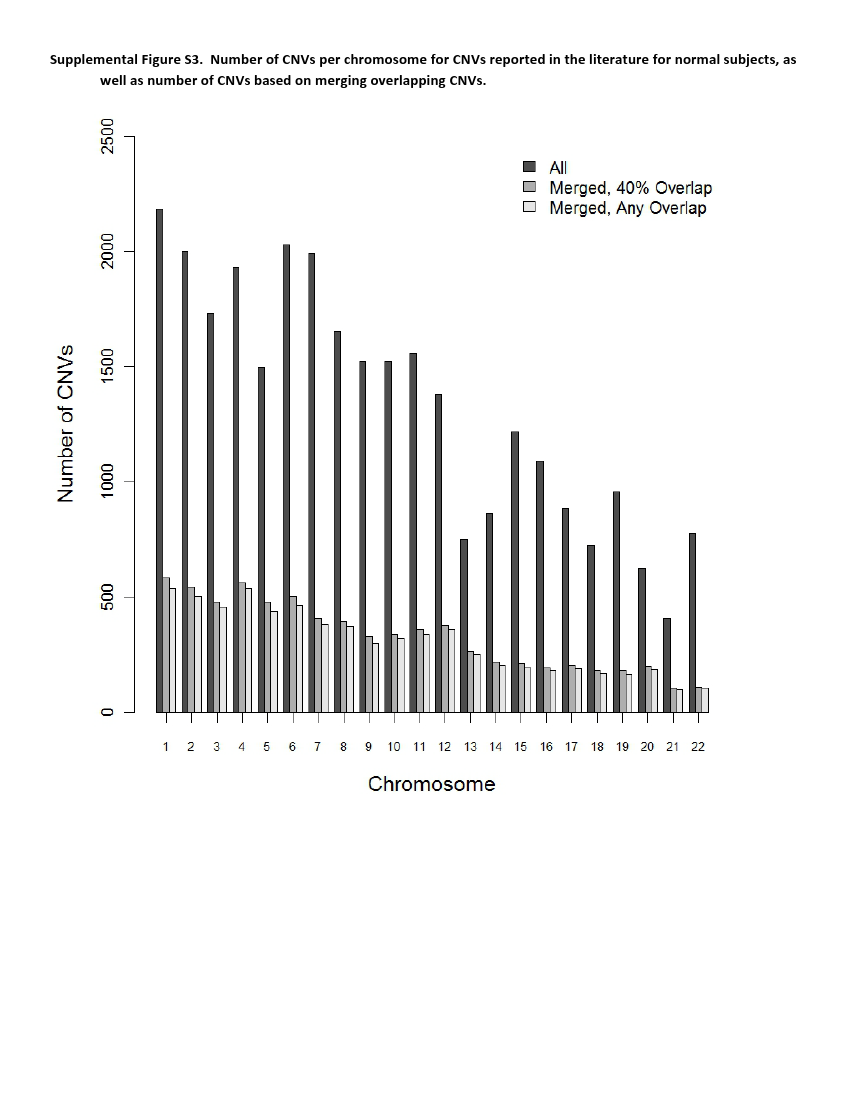

Supplement: Figure S3 — Number of CNVs per chromosome for CNVs reported in the literature for normal subjects, as well as number of CNVs based on merging overlapping CNVs. (2.80 MB TIF) [file pone.0014456.s003.tif]

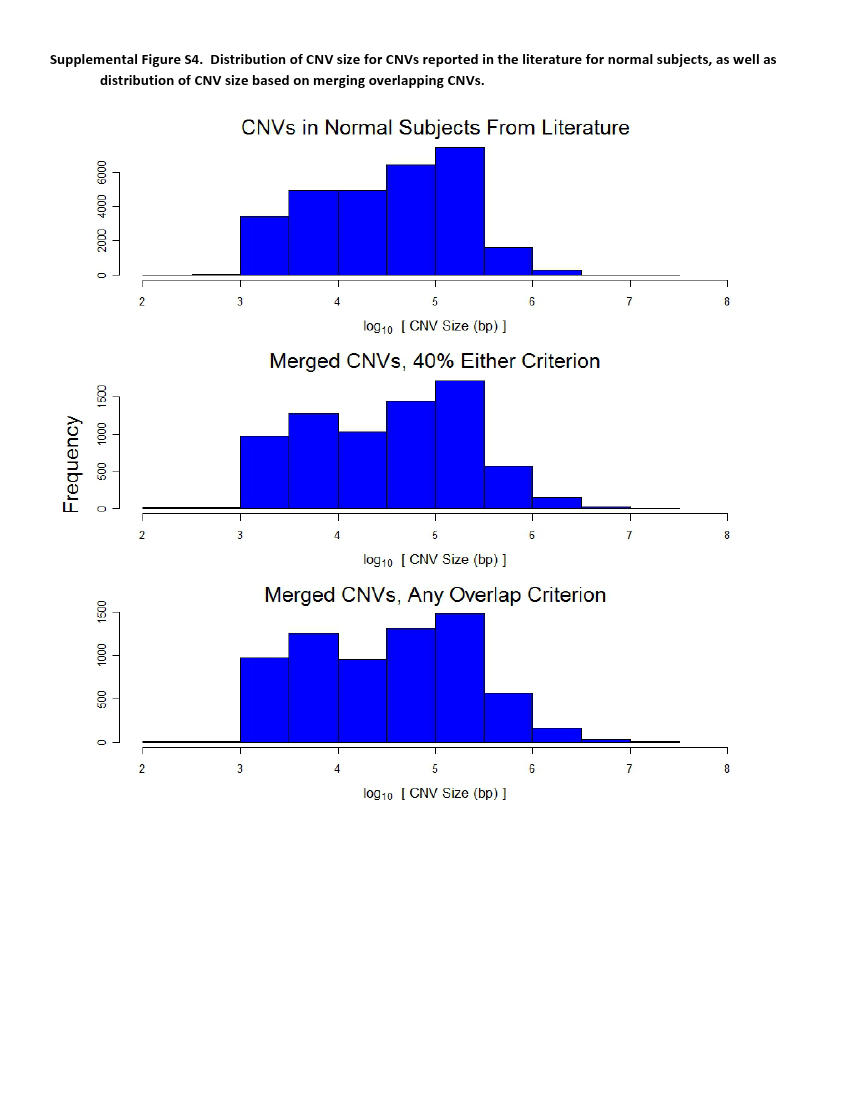

Supplement: Figure S4 — Distribution of CNV size for CNVs reported in the literature for normal subjects, as well as distribution of CNV size based on merging overlapping CNVs. (2.80 MB TIF) [file pone.0014456.s004.tif]

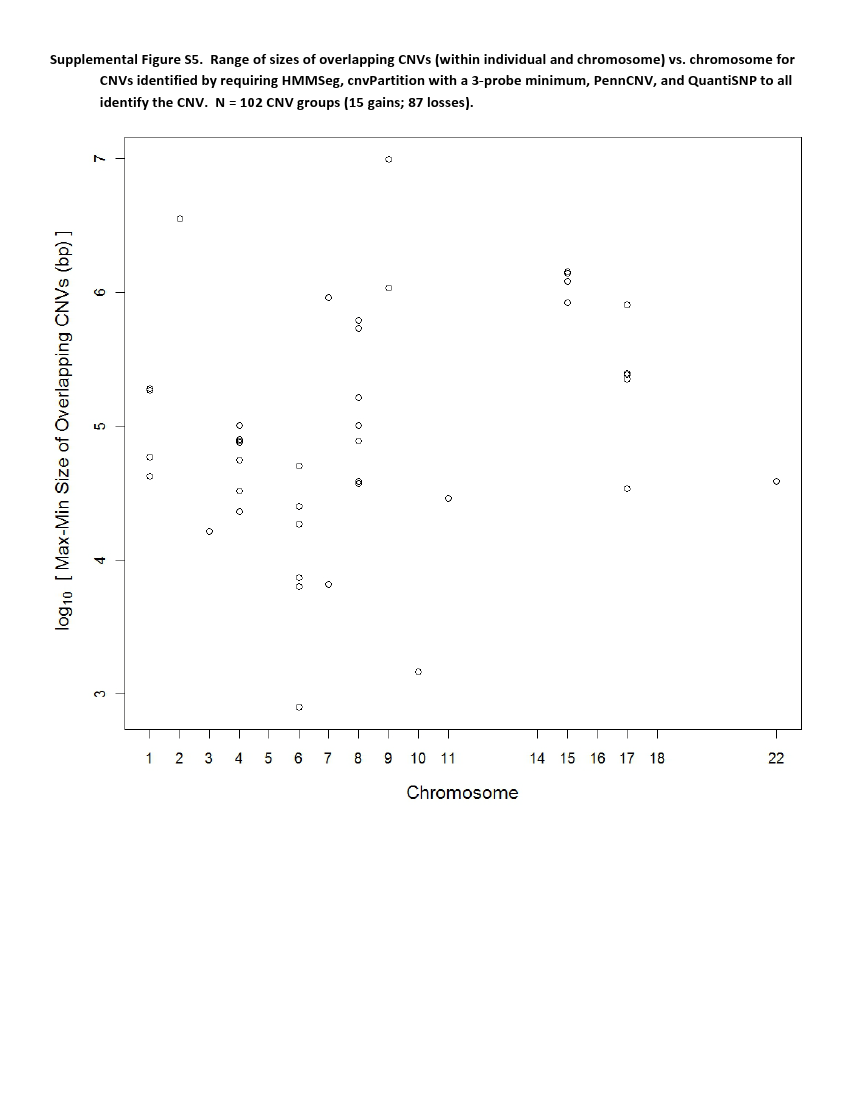

Supplement: Figure S5 — Range of sizes of overlapping CNVs (within individual and chromosome) vs. chromosome for CNVs identified by requiring HMMSeg, cnvPartition with a 3-probe minimum, PennCNV, and QuantiSNP to all identify the CNV. N = 102 CNV groups (15 gains; 87 losses). (2.80 MB TIF) [file pone.0014456.s005.tif]

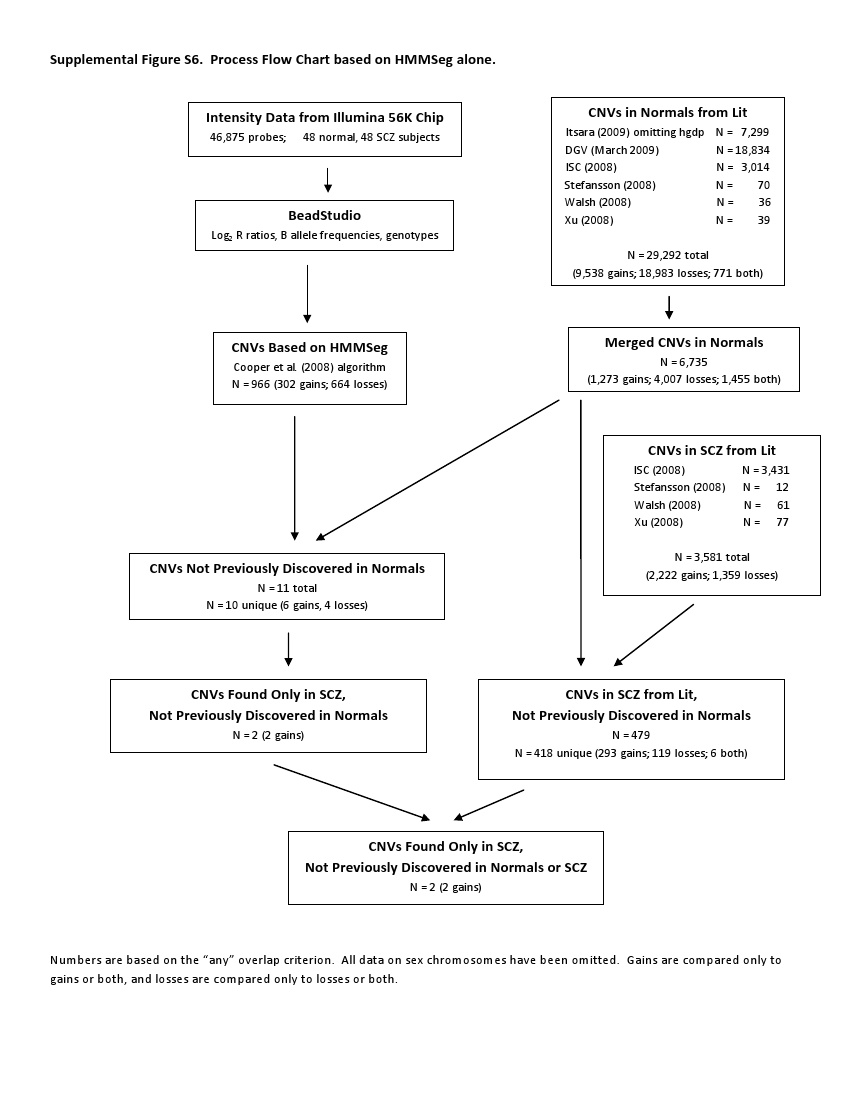

Supplement: Figure S6 — Process Flow Chart based on HMMSeg alone. (2.80 MB TIF) [file pone.0014456.s006.tif]

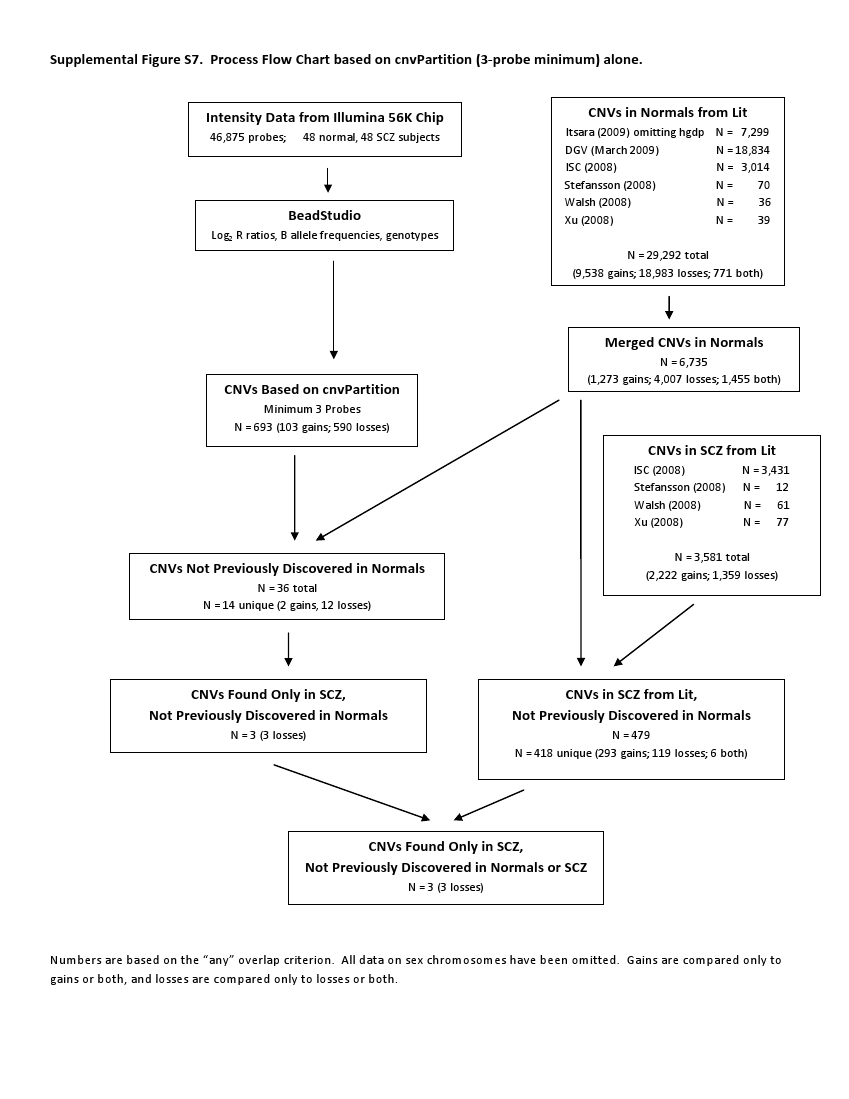

Supplement: Figure S7 — Process Flow Chart based on cnvPartition (3-probe minimum) alone. (2.80 MB TIF) [file pone.0014456.s007.tif]

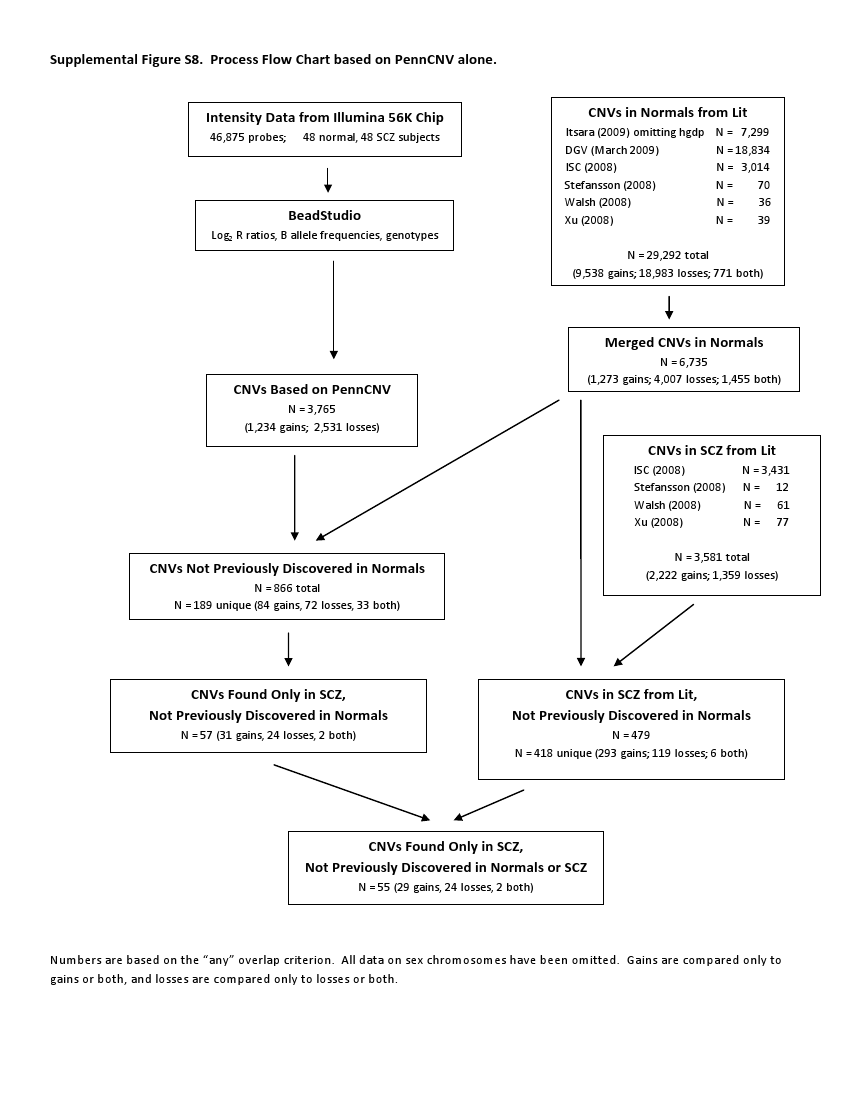

Supplement: Figure S8 — Process Flow Chart based on PennCNV alone. (2.80 MB TIF) [file pone.0014456.s008.tif]

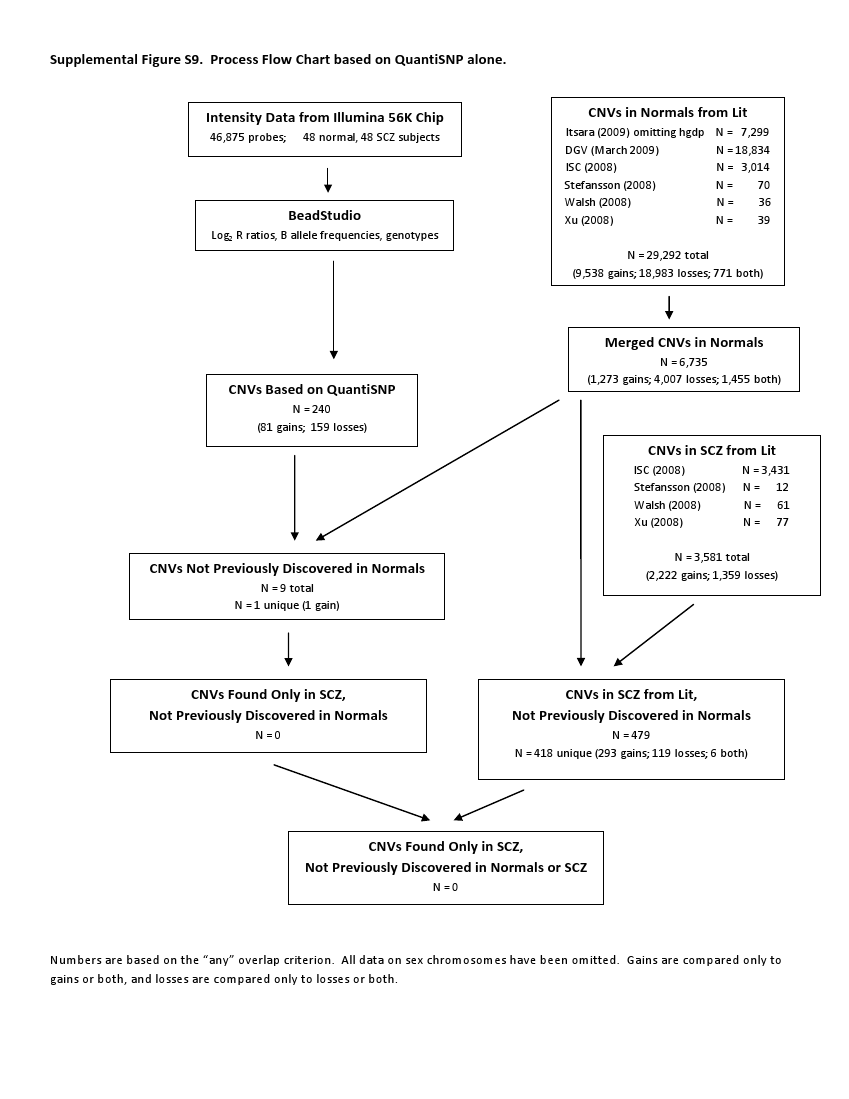

Supplement: Figure S9 — Process Flow Chart based on QuantiSNP alone. (2.80 MB TIF) [file pone.0014456.s009.tif]

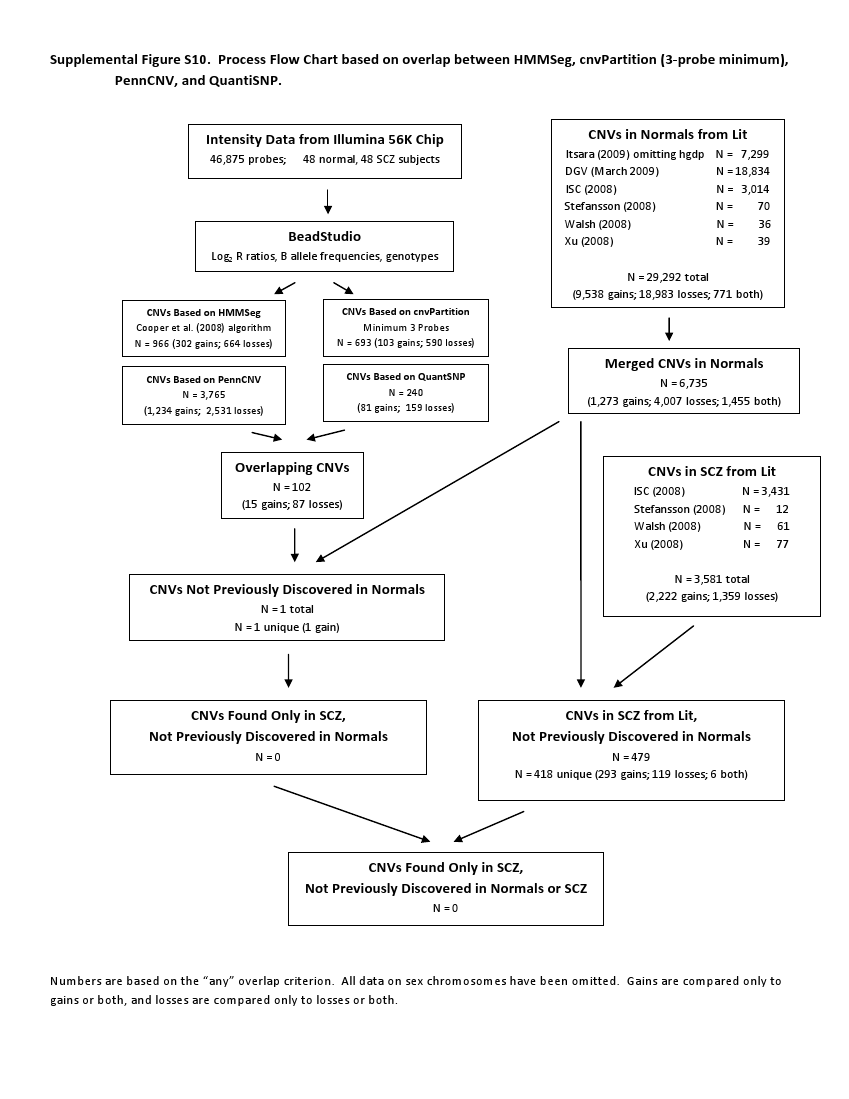

Supplement: Figure S10 — Process Flow Chart based on overlap between HMMSeg, cnvPartition (3-probe minimum), PennCNV, and QuantiSNP. (2.80 MB TIF) [file pone.0014456.s010.tif]
